# Magnetostructural Transition in Spin Frustrated Halide Double Perovskites

**DOI:** 10.1021/acs.chemmater.5c00610

**Published:** 2025-09-08

**Authors:** Kunpot Mopoung, Quanzheng Tao, Fabio Orlandi, Kingshuk Mukhuti, Kilian S. Ramsamoedj, Utkarsh Singh, Sakarn Khamkaeo, Muyi Zhang, Maarten W. de Dreu, Elvina Dilmieva, Emily L. Q. N. Ammerlaan, Thom Ottenbros, Steffen Wiedmann, Andrew T. Boothroyd, Peter C. M. Christianen, Sergei I. Simak, Johanna Rosen, Feng Gao, Irina A. Buyanova, Weimin M. Chen, Yuttapoom Puttisong

**Affiliations:** † Department of Physics, Chemistry, and Biology (IFM), 4566 Linköping University, SE-58183, Linköping, Sweden; ‡ ISIS Neutron and Muon Source, Science and Technology Facilities Council, Rutherford Appleton Laboratory, Harwell Campus, Didcot Oxfordshire, OX11 0QX, Oxford, United Kingdom; § High Field Magnet Laboratory (HFML - EMFL), 6029 Radboud University, Toernooiveld 7, 6525 ED, Nijmegen, The Netherlands; ∥ Department of Physics, Clarendon Laboratory, Oxford University, OX1 3PU, Oxford, United Kingdom; ⊥ Department of Physics and Astronomy, Uppsala University, SE-75120, Uppsala, Sweden

## Abstract

Geometrical frustration in the face-centered-cubic (fcc) lattice presents a fundamental challenge in determining antiferromagnetic order, as the ground state is highly sensitive to subtle differences in competing magnetic interactions and structural symmetry. Here, we explore the magnetostructural interplay in two halide double perovskites, Cs_2_NaFeCl_6_ and Cs_2_AgFeCl_6_. Although both materials have a cubic structure at room temperature, neutron diffraction shows that they adopt different antiferromagnetic structures upon cooling. Cs_2_NaFeCl_6_ experiences a transition to an AFM-III order below 2.6 K, governed by *J*_1_ and *J*_2_ (first and second nearest-neighbor) magnetic exchange interactions. Cs_2_AgFeCl_6_, however, adopts an AFM-I order below 17 K, accompanied by a significant tetragonal distortion confirmed from both neutron diffraction and polarized Raman spectroscopy. Thermal expansion measurements reveal anomalous lattice expansion at the magnetic transitions in both compounds but are substantially stronger in Cs_2_AgFeCl_6_. Combining these findings with density functional theory (DFT) studies, we conclude that the strength of magnetoelastic coupling dictates the magnetic ground state. A strong *J*_1_ in Cs_2_AgFeCl_6_ induces a large tetragonal lattice distortion, relieving magnetic frustration and stabilizing the AFM-I phase. In contrast, weaker magnetoelastic coupling in Cs_2_NaFeCl_6_ causes minimal distortion, favoring the AFM-III phase via the *J*_1_–*J*_2_ mechanism. Our findings show that magnetic interactions can be a primary driving force for structural phase transitions in these materials, while the strong structural distortion could determine the selection of magnetic ground-state ordering.

## Graphical abstract

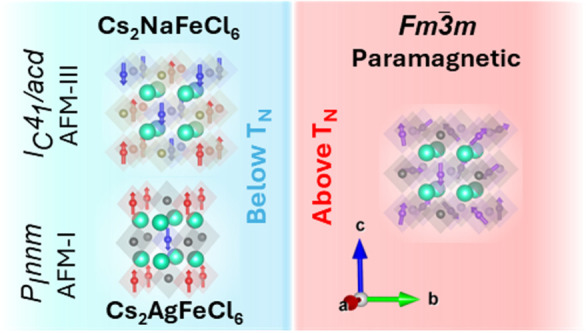

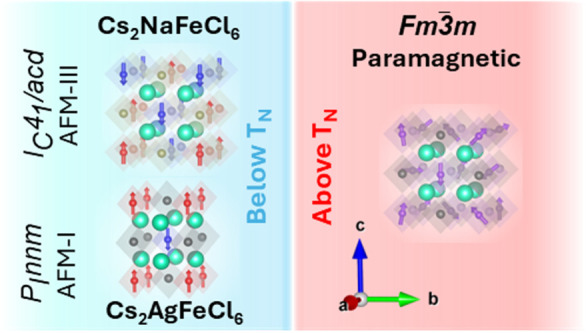

## Introduction

Halide double perovskites (HDPs) are gaining significant attention as environmentally friendly, lead-free materials that can potentially replace traditional lead-based metal-halide perovskites.^1–12^ Their crystal stability and tunable electronic properties make them suitable for various optoelectronic applications such as solar cells, LEDs, and photodetectors.^4^ These materials have the general formula *A*_2_ *BB*′*X*_6_, where *A* is an alkali metal cation, *B* and *B*′ are monovalent and trivalent metal cations, respectively, and *X* is a halogen anion. The HDPs present significant advantages over traditional metal halide perovskites (MHPs) by addressing key limitations of the latter such as their inherent instability and the toxicity associated with lead-containing compositions. HDPs, often exhibiting superior thermal, moisture, and light stability due to their typically all-inorganic nature and the substitution of lead with less toxic elements, offer a pathway to more durable and environmentally friendly devices.^2–4^ Furthermore, the *A*_2_ *BB*′*X*_6_ structure of HDPs provides greater chemical and structural versatility as compared to the *ABX*_3_ structure of MHPs, allowing for the incorporation of a wider range of metal cations that enables the tuning and tailoring of electronic, optical, and potentially magnetic properties for diverse applications,^4–6,11,12^ including spintronics.^7–10^ While the optoelectronic performance of HDPs currently lags that of top-performing MHPs, their enhanced stability, reduced toxicity, and potential for novel functionalities make them a promising class of materials for future optoelectronics such as in white-lighting^1^ and spintronic technologies.^7–10^ The ability to incorporate magnetic ions at the *B*′ site introduces exciting possibilities for magnetic functionality in HDPs.^5,8,9^ The recent discovery of long-range antiferromagnetic (AFM) ordering in HDPs^8,9^ has stimulated further interest in these materials, particularly for spintronics applications.

Beyond the scope of potential applications, AFM-HDPs also serve as excellent model systems for studying the intriguing physics of magnetic frustration.^13–18^ This frustration arises from the B′-site order of magnetic ions within the cubic crystal structure, preventing the simultaneous satisfaction of all antiferromagnetic interactions; see Figure 1A,B. In Fe-based HDPs like Cs_2_(Na/Ag)­FeCl_6_, the Fe^3+^ ions at the *B*′ sites form a face-centered cubic (fcc) network with four interpenetrated magnetic sublattices. This geometry leads to magnetic frustration, which results in a degeneracy of different AFM ground states, ranging from collinear and coplanar arrangements to incommensurate orderings.^15,19^ Understanding the factors that govern the selection of a specific ground state is crucial, as it can directly influence the material’s magnetic, optical, and transport properties.

**Figure 1:**
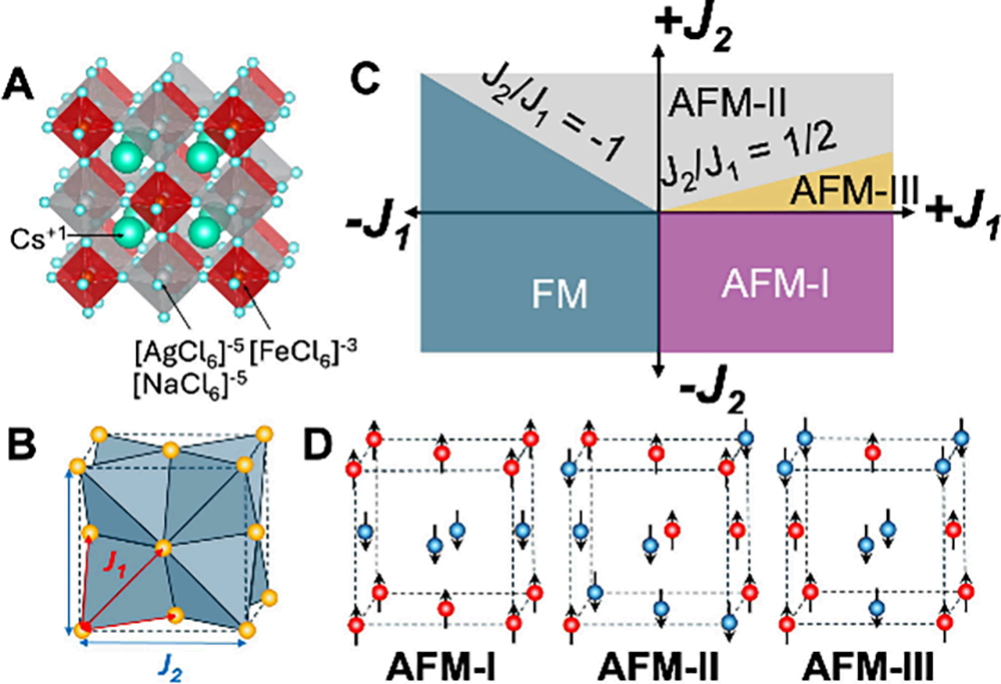
**A** Crystal structure of the halide double perovskites Cs_2_AgFeCl_6_ and Ca_2_NaFeCl_6_ at 300 K_._ **B** The Fe^3+^ magnetic lattice arranges in a face-centered cubic configuration, forming the spin frustrated network for antiferromagnetism. *J*_1_ is the exchange interaction between the nearest-neighbor magnetic ions. *J*_2_ is the exchange interaction between the next-nearest neighbors. **C** A schematic phase diagram based on the *J*_1_–*J*_2_ model. **D** Schematic of the magnetic-ion arrangement of the different colinear AFM phases (AFM-I, AFM-II and AFM-III).

This study focuses on identifying the factors that determine the magnetic ordering in two structurally similar AFM-HDPs: Cs_2_NaFeCl_6_ and Cs_2_AgFeCl_6_. By employing neutron diffraction, Raman scattering, dilatometry, and magnetization techniques, we investigate the role of the magnetoelastic coupling in selecting the antiferromagnetic ground state. This investigation aims to establish the fundamental principles for designing AFM-HDPs tailored to target applications in spintronics and multiferroic materials as well as to contribute to the understanding of fcc antiferromagnets within the framework of the three-dimensional Heisenberg model.

Our study distinguishes itself from recent investigations of spin frustration in cubic vacancy-order K_2_IrCl_6_ ^19^ and cubic oxide double perovskites (ODPs),^20^ such as Ba_2_YRuO_6_ and Ba_2_LuRuO_6_. While these materials exhibit similar fcc antiferromagnet arrangements, the underlying magnetic exchange interactions differ significantly. In K_2_IrCl_6_, despite the fcc arrangement of magnetic ions with Cl-octahedral coordination, the strong spin–orbit coupling of Ir^4+^ introduces a significant Kitaev term in the spin exchange Hamiltonian. This results in the stabilization of the AFM-III magnetic structure, which remains insensitive to changes in external perturbations. The ODPs exhibit both Kitaev and biquadratic magnetic exchange interactions, leading to non-coplanar spin arrangement stabilization.

Our chosen HDPs, Cs_2_NaFeCl_6_ and Cs_2_AgFeCl_6_, exhibit only the Heisenberg type of exchange interaction, which in turn brings the magnetic system into a region with a near-degenerate ground state. This is due to the 3d^5^ electron configuration of the Fe^3+^ ions, where each *e*_*g*_ and *t*_2*g*_ d-orbital is singly occupied. This electronic configuration effectively quenches the spin–orbit interaction, leading to negligible Kitaev interactions and other higher-order interactions. Our previous first-principles calculations confirmed that the magnetic behavior of these systems can be accurately described by the 3D Heisenberg model^9,13,14,16^

$$H=+\overset{nn}{\underset{{\left\langle i,j\right\rangle }^{\prime }}{\sum }}J_{1}S_{i}S_{j}+\overset{nnn}{\underset{{\left\langle n,m\right\rangle }^{\prime }}{\sum }}J_{2}S_{n}S_{m}+\underset{i}{\sum }K{S_{z}}^{2}$$ 1

where the first term describes the interaction between each spin sublattice *i* and its nearest neighbor (nn) spins *j* with an exchange interaction parameter *J*_1_. This parameter takes a positive (negative) value for antiferromagnetic (ferromagnetic) coupling following the standard convention. The second term describes the interaction between the next-nearest neighbors (nnn), characterized by the exchange parameter *J*_2_, summed over *n*,*m* sites. The last term accounts for the single-ion anisotropy, characterized by the anisotropy constant *K*, arising from the crystal field effect. *K* becomes negligible for 3d^5^ ions in a cubic field environment but can be decisive in the selection of the magnetic ground state when the crystal symmetry is reduced.

Equation 1 implies that, when the ratio of *J*_2_/*J*_1_ approaches zero and *K* is negligible, the ground state of the fcc magnet becomes highly degenerate. Consequently, even small perturbations can influence the selection of the magnetic ground state. In the absence of other spin interaction terms, various collinear arrangements, commonly known as AFM-I, AFM-II, and AFM-III, can form depending on subtle imbalances in the interactions within the magnetic sublattices. The well-adopted *J*_1_–*J*_2_ phase diagram^13,15^ for the simple Heisenberg model is schematically shown in Figure 1C, together with the geometric arrangements of the magnetic ions in the AFM-I, AFM-II, and AFM-III phases in Figure 1D.

In our previous study of Cs_2_(Na/Ag)­FeCl_6_ alloys^9^ we showed that the long-range antiferromagnetic order is mediated by superexchange interactions. The AFM transition temperature (*T*_*N*_) and the Curie–Weiss temperature (θ_CW_) depend on the choice of the *B* cation that affects the orbital hybridization of the HDPs. For Cs_2_AgFeCl_6_ and Cs_2_NaFeCl_6_ we found *T*_*N*_ = 17 and 2.6 K, respectively, and θ_CW_ = −210 and −27 K. Both Cs_2_AgFeCl_6_ and Cs_2_NaFeCl_6_ were grown in-house by the hydrothermal method which is described in the Materials and Methods section.

## Neutron Diffraction Study

To investigate the structural and magnetic configurations across *T*_*N*_, we performed temperature-dependent neutron powder diffraction (NPD) experiments. The NPD patterns of Cs_2_NaFeCl_6_ and Cs_2_AgFeCl_6_ at 30 and 1.5 K are shown in Figure 2A–D. Above *T*_*N*_ (at 30 K) they are well fitted with structure-only peaks, yielding a cubic *Fm*3̅*m* structure with lattice parameters of *a* = 10.2138(5) Å (Cs_2_AgFeCl_6_) and 10.277(5) Å (Cs_2_NaFeCl_6_).

**Figure 2:**
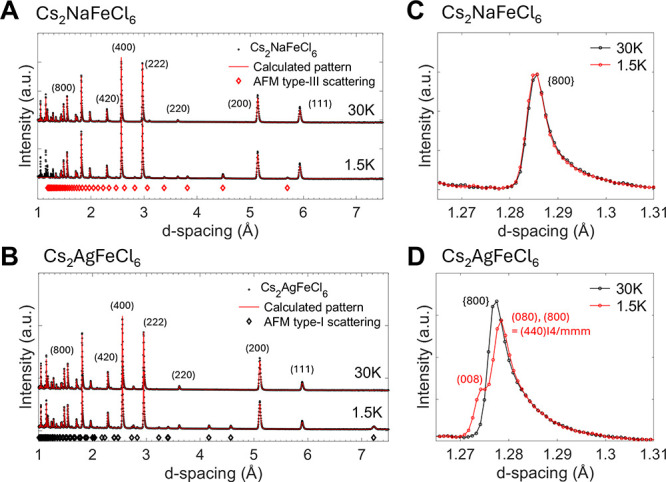
**A, B** NPD spectra at 1.5 and 30 K for Cs_2_NaFeCl_6_ (**A**) and Cs_2_AgFeCl_6_ (**B**) collected on the WISH diffractometer bank with average 2θ = 58.3°. The red curves are the fitting results, where each (*hkl*) crystallography peak is marked. The magnetic diffraction peaks, that appeared at 1.5 K, are marked with the red diamonds. **C, D** Close-up view of the {800} peaks for both samples collected by the back scattering detector bank (2θ = 152.8°). For Cs_2_AgFeCl_6_ at 1.5 K, a peak splits indicating a lowering of the crystal symmetry to tetragonal is observed. Symmetry analysis suggests a nuclear phase transition from *Fm*3̅*m* to *I*4/*mmm*, redefining the observed peaks around 1.28 Å as the {440} and (008) peaks in a tetragonal unit cell.

Below *T*_*N*_ new sets of diffraction peaks appear in both samples, which can be assigned to magnetic diffraction. For Cs_2_NaFeCl_6_, these peaks can be indexed with the propagation vector **k** = (1/2 1 0), which is characteristic of the AFM-III configuration shown in Figure 1A. It is worth noting here that the full-width half-maximum of these reflections is slightly larger than the nuclear one (which are resolution limited), indicative of a shorter length scale for the spin correlations. This finding together with the very low transition temperature and the high value of the frustration index (θ_CW_/*T*_*N*_) indicate a significant degree of frustration in this material. The magnetic structure is described by the *I*_*c*_4_1_/*acd* magnetic space group, with the mW_3_ irreducible representation (irrep) of the parent space group with order parameter direction (α, α; 0, 0; 0, 0) and assuming the action of a single arm of the star of *k*. A careful investigation of the structural diffraction {800} peak, as shown in Figure 2C, does not indicate any splitting, therefore suggesting that the lattice remains metrically cubic upon the magnetic transition, and no atomic displacement distortions or strains are observed at least within the resolution of our data. This situation allows us only to determine if the spin direction is parallel or perpendicular to the propagation vector. The magnetic transition lowers the symmetry to the tetragonal magnetic space group *I*_*c*_4_1_/*acd* with a magnetic moment parallel to the propagation vector, and consequently, the primary magnetic order parameter will induce nuclear distortions as secondary order parameters through magnetoelastic coupling. These distortions, like the tetragonal Γ_3_^+^ strain and displacements transforming as the Γ_3_^+^ and X_4_^–^ irreps are nonzero in the ground state but below the resolution of our experiment. The Rietveld plots are shown in Figure 2A, and some selected refined parameters are reported in Table 1.

**Table 1:** Lattice Parameters Obtained from the Rietveld Refinement of the NPD Data and the Position of the Cl Site in the Na Compound at 1.5 K Given in the Cubic Space Group due to the Absence of a Measurable Lattice Distortion^a^

	Cs_2_NaFeCl_6_		Cs_2_AgFeCl_6_	
	30 K	1.5 K	30 K	1.5 K
Crystal structure	*Fm*3̅*m*	*I*_*C*_4_1_/*acd*	*Fm*3̅*m*	*P*_*I*_ *nnm*
Lattice parameter	*a* = 10.27759(5) Å	*a* = 10.27638(5) Å	*a* = 10.2138(7) Å	*a*_*I*4/*mmm*_ = 7.2285(8) Å
				*c*_*I*4/*mmm*_ = 10.1914(7) Å
Atom coordinate	Cl_1_ site 0.23254(7)	Cl_1_ site 0.23259(8)	Cl_1_ site 0.23394(8)	Cl_1_ site *x* = 0.234(4), *y* = 0.766(4), *z* = 0
Magnetic structure	Paramagnetic	AFM-III	Paramagnetic	AFM-I
μ_order_ (μ_B_/Fe)	-	3.4 ± 0.02	-	3.5 ± 0.02

a The position of the Cl site in the Ag compound is given for the tetragonal *I*4/*mmm* space group as described in the text. We do not observe any additional orthorhombic distortion due to the magnetic long-range ordering.

The situation is more complicated in Cs_2_AgFeCl_6_ (Figure 2B). Below *T*_*N*_ the magnetic diffraction peaks can be indexed with the propagation vector **k** = (0 0 1), a signature of the AFM-I magnetic ordering. Contrary to the Na compound, the magnetic reflections are resolution limited, indicating a long correlation length, which, considered together with the higher transition temperature, suggests a lower degree of frustration. The AFM-I structure is described by the mX_5_^+^ irreducible representation, and the relative magnetic space group depends on the direction of the magnetic moments within the ab tetragonal plane. Because the system remains metrically tetragonal, we are not able to determine the moment direction in the ab plane from the powder data. Therefore, we assumed the moment to point in a ⟨100⟩ direction of the cubic cell with the *P*_*I*_ *nnn* magnetic space group, which corresponds to the order parameter direction (μ_1_, μ_2_; 0, 0; 0, 0). Concomitantly with magnetic ordering, a splitting of the nuclear diffraction peaks was observed. This is evident from the analysis of the {800} peak collected by the high-resolution bank in backscattering geometry, at *d*-spacing 1.27–1.29 Å, which is enlarged in Figure 2D. Below *T*_*N*_ the reflection peak splits into two peaks with a 2:1 ratio, characteristic of a cubic-to-tetragonal phase transition. The ISODISTORT software^21^ was used to investigate the possible nuclear subgroups, i.e., *I*4/*mmm*, *Fmmm* and *Immm*. The best agreement with the least number of refinable parameters was obtained with the *I*4/*mmm* space group corresponding to the action of the Γ_3_^+^ irreducible representations with the order parameter direction (δ_1_, 0). The *I*4/*mmm* structure is defined in a unit cell with $a_{I4/mmm}=\frac{a}{\sqrt{2}}$ and *c*_*I4/mmm*_ = c, redefining the scattering plane (080), (800) into (440)_*I*4/*mmm*_, as marked in Figure 2D. This therefore yields *c*_*I*4/*mmm*_ = 10.1914(7) Å and *a*_*I*4/*mmm*_ = 7.2285(8) Å. At 1.5 K the structural distortion mainly induces a change in the Ag coordination environment and produces two short Ag–Cl bonds along the tetragonal *c*-axis and four long ones in the *ab*-plane. The crystal structural parameters obtained from the Rietveld refinement are given in Table 1, and the corresponding Rietveld plot is shown in Figure 2. It is worth stressing that a linear quadratic coupling term in the system free energy exists between the magnetic order parameter and the structural one (details in the Supporting Information and in the Symmetry Analysis of Magnetic Alignment Based on Neutron Powder Diffraction Data section). This coupling invariant suggests that the structural distortion transforming as the Γ_3_^+^ irreducible representation could select the mX_5_^+^ AFM-I ground state and, in this way, reduce the system free energy and the frustration in the magnetic interactions. This could explain the order of magnitude increase in the transition temperature between the Na and Ag compounds, as well as the different magnetic structures observed in the two compounds.

## Polarized Raman Study for the Structural Phase Transition

The NPD data suggest that the tetragonal structural phase transition plays a role in determining the AFM ground state. To establish this correlation and to provide a complementary probe for the structural phase transition across *T*_*N*_, we employed polarized Raman spectroscopy. This technique allows us to detect changes in the crystal structure through the Raman selection rules, thereby probing the low-temperature crystal phase of both compounds.

The geometry of the linear polarization mapping experiments, also known as polarization-orientation (PO) Raman scattering,^22^ is schematically shown in Figure 3A. Here, a 661 nm laser with linear polarization generates Raman scattering signals that were detected in a backscattering configuration from the (111) crystal plane (the sample surface). Azimuthal dependences of the Raman signals were measured by rotating the linear polarization direction of the excitation light by an angle θ from 0 to 2π in the (111) plane. The detection polarization of the Raman signal was chosen to be either parallel (∥) or perpendicular (⊥) to the excitation polarization, corresponding to co-rotation mapping with angle θ or with θ + 90°, respectively. In a PO-Raman analysis, the angular dependence of the Raman intensity in any given configuration is proportional to |*JRJ*′|^2^, where *J* is the Jones birefringence tensor related to the symmetry of the optical selection rules and *R* is the Raman tensor of a given mode symmetry. Therefore, the analysis of the PO-Raman pattern evaluates the structural symmetry via Raman tensor *R* and the degree of linear polarized light response via Jones tensor *J*.

**Figure 3:**
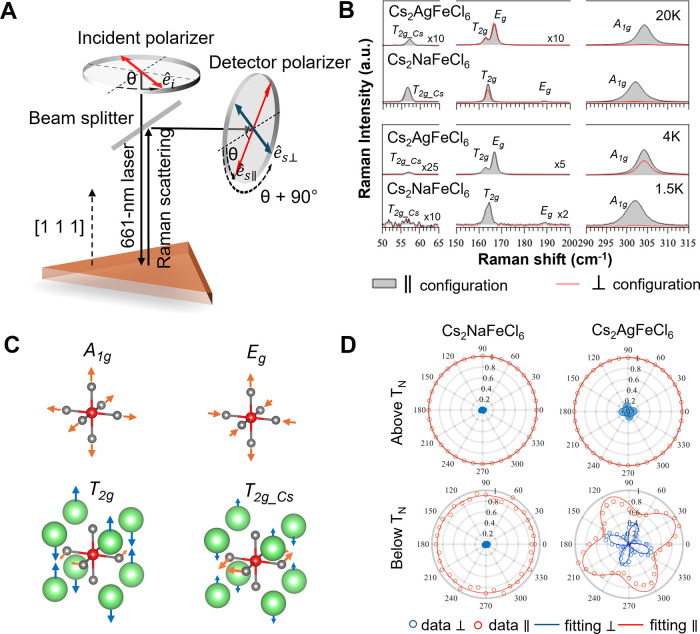
**A–D** Schematic illustration of the experimental setup for the polarization orientation (PO) Raman technique used to probe the crystal symmetry response near the AFM phase transition. **B** The total sum of the Raman scattering signal in the parallel and perpendicular configurations above and below *T*_*N*_ for Cs_2_AgFeCl_6_ and Cs_2_NaFeCl_6_. **C** Vibration modes of the *Fm*3̅*m* structure. **D** Azimuthal polarization mapping of the Raman signal of the *A*_1*g*_ mode along the ⟨111⟩ crystallography back scattering detection in both the parallel and perpendicular configurations. The measurements were performed both above *T*_*N*_ (at 20 K) and below *T*_*N*_ (at 4 K for Cs_2_AgFeCl_6_ and 2.3 K for Cs_2_NaFeCl_6_). Here the circular pattern indicates cubic symmetry and the flower-like pattern a tetragonal symmetry.

The total sum ( $\overset{2\pi }{\underset{\theta =0}{\sum }}I_{Raman}$ ) over the azimuthal angle θ of the Raman scattering signal in the parallel (gray shaded line) and perpendicular (red line) configuration above and below *T*_*N*_ is plotted in Figure 3B. Above *T*_*N*_, Raman modes from both Cs_2_NaFeCl_6_ and Cs_2_AgFeCl_6_ samples are derived from a cubic symmetry, which is assigned as *A*_1*g*_ + *E*_*g*_ + *T*_2*g*_ + *T*_2*g*_*Cs*_ following from the irreducible representation of the system point group.^11,23^ Here *T*_2*g*_*Cs*_ is the external translational mode of the Cs^+^ lattice, and *A*_1*g*_, *E*_*g*_, and *T*_2*g*_ are the internal modes of the octahedra as depicted in Figure 3C. The peak assignments in Figure 3B are based on the PO-Raman selection rules, that is, *I*_*E*_*g*_⊥_/*I*_*E*_*g*_∥_ = 1, *I*_*T*_2*g*_⊥_/*I*_*T*_2*g*_∥_ = 2/3 and *I*_*A*_1*g*_⊥_/*I*_*A*_1*g*_∥_ = 0.

Below *T*_*N*_, a breakdown of the cubic Raman selection rule is clear in Cs_2_AgFeCl_6_. A prominent feature appeared at the *A*_1*g*_ peak, where the Raman selection rule relaxes to allow Raman detection in the ⊥ configuration, yielding a *I*_*A*_1*g*_⊥_/*I*_*A*_1*g*_∥_ ratio as large as 0.7. For the other Raman peaks, the intensity ratio also changes but in a more subtle way. In principle, lowering of the crystal symmetry also lifts the degeneracy of the *T*_2*g*_*Cs*_, *T*_2*g*_ and *E*_*g*_ modes, leading to the splitting of the Raman peaks into modes of lower symmetry. Such effects were not observed in our spectra, which is attributed to a rather small energy splitting of the Raman modes as compared to the line width such that it cannot be spectrally resolved in our experiment. Nonetheless, the Raman selection rule of the *A*_1*g*_ mode provides us with a tool for determining the crystal phase.

To determine the exact symmetry of the crystals above and below *T*_*N*_, we analyze the azimuthal mapping of the PO-Raman signal of the *A*_1*g*_ mode, which is plotted in Figure 3D. For the Cs_2_NaFeCl_6_ sample, the PO-Raman pattern of the *A*_1*g*_-mode exhibits no angular dependence in the ∥ configuration, whereas it vanishes in the ⊥ configuration, following the selection rules for cubic symmetry both above and below *T*_*N*_. This suggests that no substantial lattice distortion occurs in this material below the magnetic phase transition temperature, consistent with the NPD results of Figure 2C. In contrast, the PO-Raman patterns of Cs_2_AgFeCl_6_ match with a cubic-to-tetragonal transition. It transforms into a 4-fold flower pattern, indicative of tetragonal symmetry. This tetragonal structure is confirmed by a detailed analysis fitting, as described in the Supporting Information, where both the Raman and Jones tensors (*R* and *J*) exhibit characteristics of tetragonal symmetry.

The polarized Raman spectroscopy results confirm a structural transition in Cs_2_AgFeCl_6_. To establish how a cubic-to-tetragonal phase transition relates to the AFM-I ordering (in contrast to the AFM-III ordering without visible structural change in Cs_2_NaFeCl_6_) we compared the lattice distortion and magnetic ordering (*M*_order_) obtained by Raman and NPD measurements.

In the NPD data in Figure 4A, the refinement yielded the lattice parameters and the ordering magnetic moment per Fe atom (μ_order_) as a function of the temperature. The results clearly demonstrate a strong correlation between structural symmetry and AFM-I magnetic ordering. Above *T*_*N*_ (>19 K), Cs_2_AgFeCl_6_ is in a paramagnetic state with zero net magnetic moment. The lattice parameter maintains a constant cubic value of *a* = 10.2138(7) Å. Below *T*_*N*_, a continuous rise in μ_order_ concurrent with an increasing tetragonal distortion, characterized by the lattice parameters *c* and *a* (which are *c*_*aI*4/*mmm*_ and $\sqrt{2}a_{I4/mmm}$ in the tetragonal lattice cells). The maximum ordered moment at 1.5 K is μ_order_ = 3.50 ± 0.02 μ_B_/Fe. We note that the μ_order_ value is lower than that of the free ions (5 μ_B_/Fe) which is common in solids, due to the combined effect of crystal field and covalence effects.

**Figure 4:**
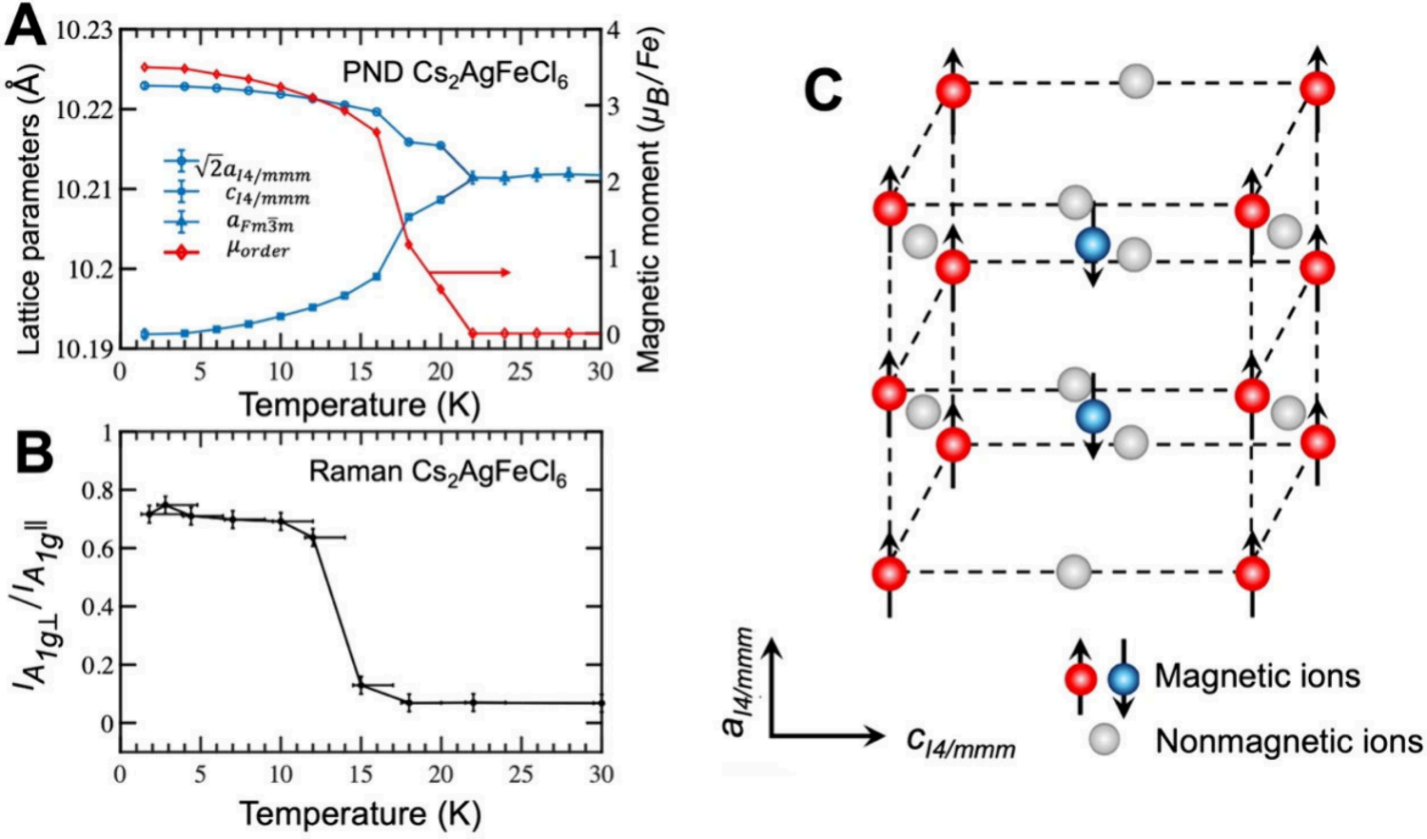
**A** Temperature-dependent fitting parameters for Cs_2_AgFeCl_6_. Blue triangles represent cubic structure (*Fm*3̅*m*) lattice parameters, while blue circles and rectangles correspond to tetragonal structure parameters (*I*4/*mmm*). The red line represents the ordering magnetic moment per Fe atom. **B** Temperature dependence of *I*_*A*_1*g*_⊥_/*I*_*A*_1*g*_∥_ for Cs_2_AgFeCl_6_. The error bar in temperature is due to local laser heating. **C** The AFM-I ordering within a tetragonal unit cell.

In the Raman experiments shown in Figure 4B, we use the *I*_*A*_1*g*_⊥_/*I*_*A*_1*g*_∥_ ratio as a probe of the crystal phase transition in Cs_2_AgFeCl_6_ and correlate the trend with the tetragonal distortion obtained from NPD data. This correlation is rather striking, and the *I*_*A*_1*g*_⊥_/*I*_*A*_1*g*_∥_ ratio develops concomitantly with the lattice distortion. Cs_2_NaFeCl_6_, on the other hand, exhibits a nearly constant *I*_*A*_1*g*_⊥_/*I*_*A*_1*g*_∥_ value, in agreement with the absence of sizable tetragonal distortion across *T*_*N*_; see Figure S1. With the structure analysis confirmed by both the NPD and Raman results, the AFM-I ordering for Cs_2_AgFeCl_6_ is schematically drawn in Figure 4C.

## The Lattice Expansion Anomaly and the Magnetostructural Transition

To resolve the apparent contradiction in the magnetostructural correlation of Cs_2_NaFeCl_6_, namely, the inability of the neutron scattering and polarized Raman techniques to detect the predicted tetragonal symmetry accompanying its AFM-III magnetic order, we performed high-resolution thermal expansion experiments using dilatometry. These measurements revealed sharp anomalies in the thermal expansion for both Cs_2_NaFeCl_6_ and Cs_2_AgFeCl_6_ at their respective magnetic transition temperatures (*T*_*N*_ = 2.6 K and *T*_*N*_ = 17 K, Figure 5A and 5B, respectively). Such features indicate that first-order structural phase transitions occur concurrently with magnetic ordering, providing evidence for magnetostructural coupling in both materials.

**Figure 5:**
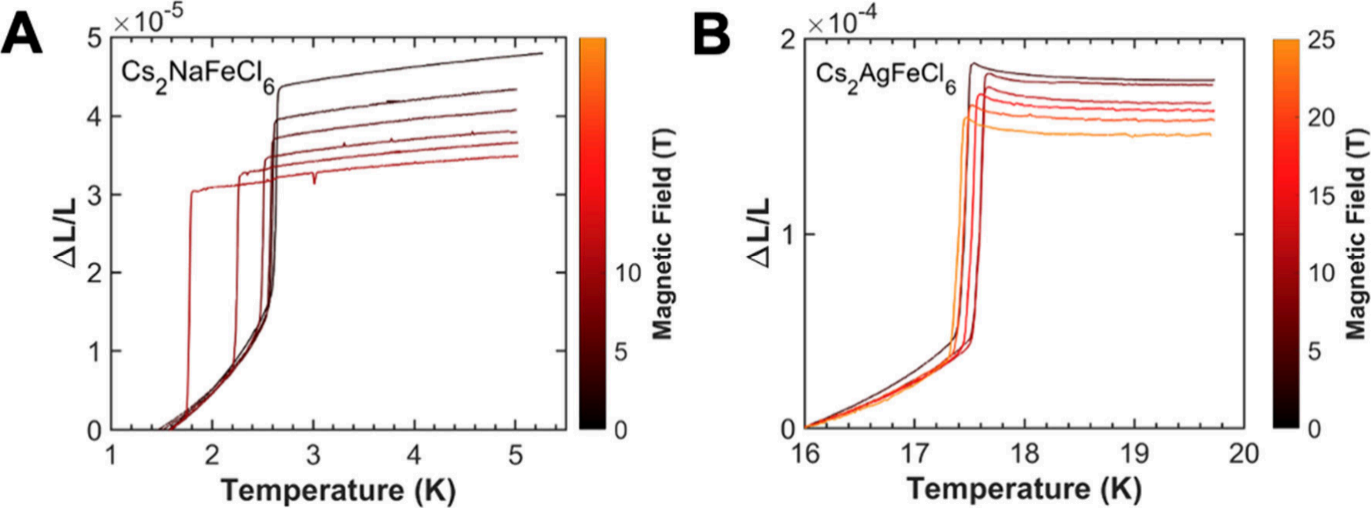
Magnetic field dependence of thermal expansion (Δ*L*/*L*) of **A** Cs_2_NaFeCl_6_ under various magnetic fields ranging from 0 to 8 T, where the sharp transitions occur around *T*_*N*_ = 1.7–2.6 K, and **B** Cs_2_AgFeCl_6_ under magnetic fields varying from 0 to 25 T, where the sharp transitions occur around *T*_*N*_ = 17.4–17.6 K.

Significantly, the magnitude of the lattice parameter change in Cs_2_NaFeCl_6_ is approximately 1 order of magnitude smaller than that in Cs_2_AgFeCl_6_. This finding explains the previous experimental challenges and directly poses the question on the selection of the different AFM ground states (AFM-III vs AFM-I), which may be correlated with the degree of structural distortion and therefore the magnetoelastic coupling. Given the low transition temperatures, which are well below the optical phonon energies,^23^ a transition driven primarily by phonon anharmonicity appears unlikely. We therefore propose that the magnetic transition itself is the primary driving force, inducing structural changes via magnetoelastic coupling and that the selection of the ground state hinges on the strength of this coupling.

To test this hypothesis and quantify the relative coupling strengths, we conducted thermal expansion experiments under high external magnetic fields. As shown in Figure 5, the *T*_*N*_ of Cs_2_NaFeCl_6_ is highly sensitive to the applied field, namely, decreasing with an increasing field. Up to 8 T, we can observe a significant shift of *T*_*N*_ from 2.6 to 1.7 K for Cs_2_NaFeCl_6_. In contrast, the *T*_*N*_ of Cs_2_AgFeCl_6_ remains robust (17.4–17.6 K) up to 25 T. This behavior indicates substantially weaker exchange interactions and magnetoelastic coupling in Cs_2_NaFeCl_6_ compared to Cs_2_AgFeCl_6_. These findings follow the previously reported Curie–Weiss temperatures^9^ (Θ_CW_) of −210 K for Cs_2_AgFeCl_6_ and −27 K for Cs_2_NaFeCl_6_, implying that Cs_2_AgFeCl_6_ has much stronger magnetic exchange interactions than that of Cs_2_NaFeCl_6_. Thus, the combined evidence suggests that the strength of the magnetoelastic coupling is a decisive factor: strong coupling in Cs_2_AgFeCl_6_ stabilizes the AFM-I order with a large distortion, while weaker coupling in Cs_2_NaFeCl_6_ favors the AFM-III order with only a minimal structural change.

## Density Functional Theory Calculation for Ground State Magnetic Ordering

To evaluate how the tetragonal strain influences the preferred magnetic ordering, we performed static DFT+U total-energy calculations for the AFM-I and AFM-III configurations in two fixed lattices: the ideal cubic cell (*c*/*a* = 1.000) and the ground state tetragonal cell (*c*/*a* = 0.987). Atomic positions were kept frozen, and the resulting energies were normalized to a per-formula-unit basis (Cs_2_AgFeCl_6_). In the cubic geometry, AFM-III is lower in energy by 2.40 meV f.u.^–1^; conversely, in the tetragonal cell AFM-I is lower by 6.48 meV f.u.^–1^. This 8.9 meV f.u.^–1^ reversal demonstrates that the modest *c*/*a* ≈ 0.99 distortion is sufficient to invert the energetic hierarchy and stabilize AFM-I as the ground state (see Table 2). Thus, the modest lattice distortion dictates the magnetic ground state, signaling the emergence of a magnetostructural coupling.

**Table 2:** DFT+U Energies of the AFM-I and AFM-III Magnetic Phases in the Cubic and Tetragonal Geometries

	*E*_AFM‑I_ (eV f.u.^–1^)	*E*_AFM‑III_ (eV f.u.^–1^)	Δ*E* (meV f.u.^–1^)
Cubic	–37.04668	–37.04908	+2.40
Tetragonal	–37.04975	–37.04327	–6.48

## AFM Ground State Selection

The observed AFM-III phase in Cs_2_NaFeCl_6_ can be described by the *J*_1_–*J*_2_ model.^12–16^ Previous density functional theory (DFT) calculations predicted that both *J*_1_ and *J*_2_ are positive (antiferromagnetic spin alignment, following eq 1) with *J*_2_/*J*_1_ ∼ 0.04, leading to the lowest-energy state to be the AFM-III phase in the *J*_1_–*J*_2_ phase diagram shown in Figure 1C, in agreement with our experimental results. Instead, the *J*_1_–*J*_2_ model would also predict AFM-III ordering in Cs_2_AgFeCl_6_ (*J*_2_/*J*_1_ ∼ 0.13), in contrast to our experimental findings. In light of the strong magnetoelastic coupling found in Cs_2_AgFeCl_6_ (and the lack thereof in Cs_2_NaFeCl_6_), we suggest that the observed AFM-I magnetic ordering is selected via the induced large magnetoelastic distortion due to strong spin–orbital hybridization along the Fe–Cl–Ag–Fe pathway, which could be parametrized via the crystal field term in the Heisenberg Hamiltonian (equation 1). We argue that the observed large tetragonal distortion relieves magnetic frustration by selecting the AFM-I magnetic ordering. A further proof of the effect of the nuclear phase transition on the magnetic structure is the change in the propagation vector between the two compounds. The choice of the **k** = (0 0 1) propagation vector in Cs_2_AgFeCl_6_ indicates that the system chooses this propagation vector to couple the magnetic order parameter ((μ_1_, μ_2_; 0, 0; 0, 0) transforming as the mX_5_ irrep) to the tetragonal distortion ((δ_1_, 0) transforming as Γ_3_^+^) and reduce the system energy through the (2δ_1_μ_1_^2^ – δ_1_μ_2_^2^) and (−δ_1_μ_1_^2^ + 2δ_1_μ_2_^2^) free energy coupling invariants (see the Supporting Information for details).

The competition between single-ion anisotropy induced by the tetragonal phase (*K*) and *J*_2_ leads to the selection between the two types of magnetic ordering. Therefore, the Na/Ag alloys are expected to further increase the degree of magnetic frustration due to the competition among these ordering types. Our previous study observed strong magnetic frustration (*f* = 12–27) in Cs_2_Ag_0.6_Na_0.4_FeCl_6_, compared to its parent compounds (*f* = 10.5–12 for Cs_2_AgFeCl_6_ and *f* = 7.7–10 for Cs_2_NaFeCl_6_).^9^ However, supplemental NPD studies of Cs_2_Ag_0.6_Na_0.4_FeCl_6_ below *T*_*N*_, as shown in Figure S3, reveal a cubic (*Fm*3̅*m*) crystal structure, like the Na-compound, but with AFM-I magnetic ordering, as observed in the Ag-compound. This scenario could not be explained by our current model and may be a result of alloy coordination randomness that plays an important role in the exchange interaction partway. Therefore, the nonmagnetic site substitution is worthy of further studies that can provide a better understanding of the correlation between magnetic and structural properties of magnetic HDPs. An optimal Ag/Na ratio may heighten the degree of magnetic frustration which could lead to the formation of the complex magnetic phases.^15,20^

## Conclusion

Our comprehensive and detailed investigations into the contrasting magnetostructural behaviors of Cs_2_NaFeCl_6_ and Cs_2_AgFeCl_6_ have uncovered a magnetostructural transition and a critical role of structural distortions in manipulating magnetic ordering in halide double perovskites. While Cs_2_NaFeCl_6_ remains metrically cubic (i.e., a subtle lattice distortion beyond instrumental resolution limit) with geometrically frustrated antiferromagnetism in the AFM-III configuration, Cs_2_AgFeCl_6_ undergoes a cubic-to-tetragonal transition under a strong magnetoelastic coupling, facilitating the relief of frustration and leading to the selection of the AFM-I configuration. The spin–orbital hybridization along the exchange interaction pathway seems to hold the key to promoting magnetoelastic coupling, which in turn leads to the selection of different AFM ground states. These results highlight the potential of utilizing orbital engineering to tailor magnetic properties in halide perovskites, paving the way for developing multifunctional magnetic materials for spintronics and information storage technologies.

## Materials and Methods

### Sample Preparation

Single crystals of Cs_2_AgFeCl_6_ and Cs_2_NaFeCl_6_ with {111} surfaces were produced through the hydrothermal method. The starting materials, consisting of CsCl, AgCl, NaCl, FeCl_3_, and HCl, were dissolved in HCl and then transferred to a Teflon-lined autoclave. The sealed autoclave was then placed in an oven and heated to 180 °C for 12 h. Finally, it was cooled down to room temperature gradually at a rate of 1 °C per hour, yielding single crystals with a lateral size of about 3–5 mm.

### Neutron Powder Diffraction

Neutron Powder Diffraction was performed at the ISIS Neutron and Muon Source, Rutherford Appleton Laboratory, STFC, Harwell Campus, Didcot., the United Kingdom, at the WISH diffractometer on the target station 2.^29^ The powder samples (ground from the single crystals) were placed in thin vanadium cans, and the diffraction data were collected in the 1.5–30 K temperature range with the support of an Oxford instrument cryostat. The raw data file of the neutron diffraction measurement can be obtained at 10.5286/ISIS.E.RB2310231.

### Polarization Orientation Raman Spectroscopy

Raman measurements were carried out using a confocal Horiba Jobin-Yon HR800 system with a 2400 l/mm single-grating monochromator fitted with a Si charge-coupled device (CCD) array. A 661 nm diode laser with a low power level of <1 mW/mm^2^ was used as an excitation source to avoid sample heating. A series of linear polarizers and λ/2 waveplates were placed along the excitation and detection paths to perform polarization-resolved experiments. The sample was cooled via a coldfinger in a He-flow cryostat. The lowest sample temperature was 4 K. Temperature-dependent experiments were conducted by adjusting the heater installed on the coldfinger.

We note that, for Cs_2_NaFeCl_6_ where the phase transition temperature is below 3.5 K, we used a wet-cryostat with He-liquid/gas in the sample chamber. This allowed us to perform Raman experiments below *T*_*N*_ at the base temperature of 1.7 K (liquid-helium-filled sample chamber, pumped) while adjusting the excitation power to as low as 0.01 mW/mm^2^ to avoid any subtle laser heating that can worsen the signal-to-noise ratio. Under this condition, the global temperature of the sample was 2.3 K. Since the 661 nm is below the absorption edge of Cs_2_NaFeCl_6_ there is no direct heat transfer to the sample by light absorption. Therefore, Cs_2_NaFeCl_6_ reached thermal equilibrium with the sample chamber at 2.3 K.

### Dilatometry

The thermal expansion measurements were conducted using capacitive dilatometry as outlined in ref 30 within a 50 mm room-temperature bore Florida-Bitter magnet at the HFML-EMFL. The relative length change, Δ*L*/*L*, where *L* is the sample length, was recorded as a function of the temperature at constant magnetic field values between 0 and 25 T. The relative length change was measured in the same direction as the applied magnetic field, which was the ⟨111⟩ direction. During all measurements, the dilatometer with the sample was placed inside a vacuum tube with an inner vacuum chamber (IVC). To ensure efficient thermal contact between the external heater (strain gauge) and the sample, ^4^He contact gas was introduced before the experiments. The temperature was varied at a controlled sweep rate of 0.5 K/min using a Lakeshore LS350 temperature controller. For each measurement, the sample was first cooled to the base temperature. Measurements were then carried out stepwise during the subsequent warming process, recording the relative length change at each temperature step.

## Supplementary Material



## Data Availability

The data and programming codes used in these studies will be provided from the corresponding authors upon reasonable request. The refined crystal structure of Cs_2_AgFeCl_6_ at 2.5 K is deposited to the CCDC database, reference number 2412060. Raw data file of the neutron diffraction measurement can be obtained at 10.5286/ISIS.E.RB2310231.

## Notes

1) Temperature-dependent polarized Raman study in Cs_2_NaFeCl_6_, 2) PO-Raman analysis, 3) the NPD analysis of Cs_2_Ag_0.6_Na_0.4_FeCl_6_, and 4) method for DFT calculation (PDF)
