# Supplementary material for: Magnetostructural Transition in Spin Frustrated Halide Double Perovskites
Source: Chem Mater. 2025 Sep 8;37(18):6974–82. doi: 10.1021/acs.chemmater.5c00610 (PMC12461833; doi:10.1021/acs.chemmater.5c00610)
Supplement: Supplementary file 1 [file cm5c00610_si_001.pdf]

Supporting Information

Magnetostructural Transition in Spin Frustrated Halide Double Perovskites

Kunpot Mopoung[a+], Quanzheng Tao[a,d+], Fabio Orlandi[b], Kingshuk Mukhuti[c], Killian S. Ramsamoedj[c], Utkarsh Singh[a], Sakarn Khamkaeo[a], Muyi Zhang[a], Maarten W. de Dreu[c], Elvina Dilmieva[c], Emily L. Q. N. Ammerlaan[c], Thom Ottenbros[c], Steffen Wiedmann[c], Andrew T. Boothroyd[d], Peter C. M. Christianen[c], Sergey I. Simak[a,e], Johanna Rosen[a], Feng Gao[a], Irina A. Buyanova[a], Weimin M. Chen[a*] and Yuttapoom Puttisong[a**]

[a]Department of Physics, Chemistry, and Biology (IFM), Linköping University, SE-58183, Linköping, Sweden

[b]ISIS Neutron and Muon Source, Science and Technology Facilities Council, Rutherford Appleton Laboratory, Harwell Campus, Didcot Oxfordshire, OX11 0QX, Oxford, United Kingdom

[c]High Field Magnet Laboratory (HFML - EMFL), Radboud University, Toernooiveld 7, 6525 ED Nijmegen, The Netherlands.

[d]Department of Physics, Oxford University, Clarendon Laboratory, OX1 3PU, Oxford, United Kingdom

[e]Department of Physics and Astronomy, Uppsala University, SE-75120 Uppsala, Sweden

* Email: weimin.chen@liu.se

** Email: yuttapoom.puttisong@liu.se

[+] These authors contribute equally to this work

Figure S1 Temperature dependence of $I_{A_{1g}\perp}/I_{A_{1g}\parallel}$ for $Cs_2NaFeCl_6$.

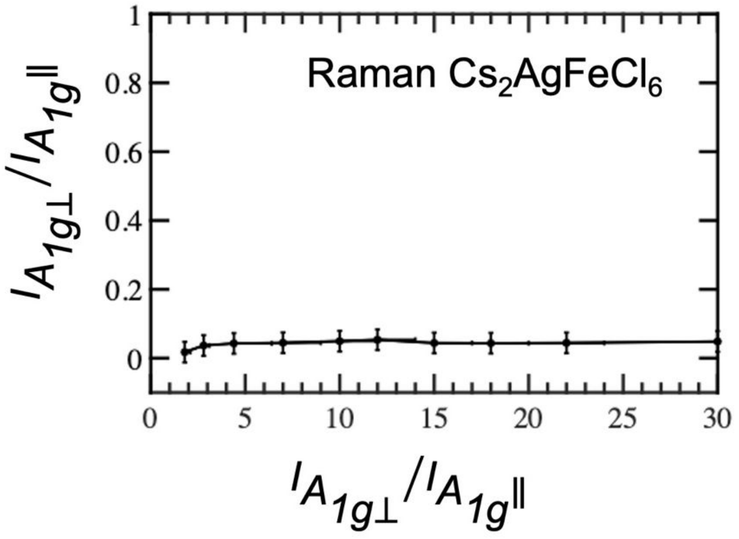

Symmetry Analysis of Magnetic Alignment Based on Neutron Powder Diffraction data

In this section, we evaluate the symmetry and correlation between the structural and the magnetic degrees of freedom. For $Cs_2NaFeCl_6$, the system remains metrically cubic below T_N and the magnetic moment direction cannot be determined directly from NPD data alone. Consequently, the magnetic moment direction is chosen to point along the propagation vector for simplicity. In this case the AFM-III magnetic structure is described by the I_c4_1/acd magnetic space group defined in a unit cell related to the parent cubic structure by the transformation matrix {(0,-1,0),(0,0,-1),(2,0,0)}, origin = (-7/4,0,1/4). This isotropy subgroup is due to the action of the mW_3 irreducible representation and with the involvement of a single arm of the star of k.

For $Cs_2AgFeCl_6$, magnetic diffraction can be indexed with the propagation vector **k** = (0 1 0). The symmetry analysis, assuming a single arm of the star of k is involved in the phase transition, suggests the magnetic ordering to transform as the mX_5^+ irreducible representation of the cubic $Fm\bar{3}m$ parent structure. In this AFM-I structure, the spins are coupled ferromagnetically in the *a-b* plane and antiferromagnetic along the *c*-axis. The metrically tetragonal unit cell allows us to determine that the magnetic moments lie in the tetragonal ab plane. The moment direction within this plane can be suggested by investigating a possible coupling between the structural distortion and the magnetic order parameter. The nuclear distortion can be described by the order parameter $(\delta_1,0)$ which transform as the Γ_3^+ irreps of the $Fm\bar{3}m$ parent space group, whereas the magnetic order parameters $(\mu_1,\mu_2;0,0;0,0)$ describe the magnetic ordering and transform as the mX_5^- irrep. It is possible to derive two free energy invariants between these two order parameters which describe their coupling $(2\delta_1\mu_1^2 - \delta_1\mu_2^2)$ and $(-\delta_1\mu_1^2+2\delta_1\mu_2^2)$. The presence of these free energy invariants suggests that the magnetic degrees of freedom could choose to order transforming as the mX_5^- irreps to couple with the lattice distortion and decrease the total energy of the system. Following the above invariants, two high symmetry order parameter directions are possible for the magnetic degree of freedom: $(\mu_1,0;0,0;0,0)$ which correspond to the magnetic space group C_Amca with moment along the <110> directions of the cubic $Fm\bar{3}m$ structure, and $(\mu_1,\mu_1;0,0;0,0)$ which correspond to the magnetic space group P_Innm with moment along the <100> direction of the $Fm\bar{3}m$ structure.

Polarization Orientation Raman spectroscopy

Polarization Orientation Raman (PO-Raman) maps out the selection rules of Raman-active modes and the linear polarization optical response tensor following the equation

$$I(\theta) \propto |e_i(\theta) J R J^{-1} e_{s\parallel,s\perp}(\theta)|^2 \quad (1)$$

where $I(\theta)$ is the Raman intensity in each rotation angle θ, J is the Jones matrix related to linear optical birefringence effects, and R is the Raman tensor. Both J and R are projected to the (111) crystal plane. $e_i(\theta)$ is the electric field vector in the (111) plane and $e_{s\parallel,s\perp}$ is the detecting polarization direction, with $e_{s\parallel}(\theta) = e_i(\theta)$ for the parallel configuration (‖) and $e_{s\perp}(\theta) = e_i$ $(\theta + 90°)$ for the perpendicular (⊥) configuration.

The form of the Jones linear birefringence tensor, which is caused by the anisotropy of tetragonal symmetry is

$$J = \begin{pmatrix} 1 & 0 & 0 \\ 0 & e^{i\gamma} & 0 \\ 0 & 0 & 1 \end{pmatrix}$$

where γ is the phase difference between the light in the fast- and slow-axis polarization.

For cubic symmetry, the Raman tensor holds the form

$$R(A_{1g}) = \begin{pmatrix} a & 0 & 0 \\ 0 & a & 0 \\ 0 & 0 & a \end{pmatrix}, R(E_{g1}) = \begin{pmatrix} -\sqrt{3}b & 0 & 0 \\ 0 & \sqrt{3}b & 0 \\ 0 & 0 & 0 \end{pmatrix}, (E_{g2}) = \begin{pmatrix} b & 0 & 0 \\ 0 & b & 0 \\ 0 & 0 & -2b \end{pmatrix}$$

$$R(T_{g1}) = \begin{pmatrix} 0 & 0 & 0 \\ 0 & 0 & d \\ 0 & d & 0 \end{pmatrix}, R(T_{g2}) = \begin{pmatrix} 0 & 0 & d \\ 0 & 0 & 0 \\ d & 0 & 0 \end{pmatrix}, R(T_{g3}) = \begin{pmatrix} 0 & d & 0 \\ d & 0 & 0 \\ 0 & 0 & 0 \end{pmatrix}$$

To fit the PO-Raman Pattern of the A_{1g}-mode, we need to use both the J and R term. Supplemental **Fig. S2** shows that the J-term contributes to the four-fold flower-like pattern, while the tetragonal symmetry of the A_{1g}-mode Raman tensors cause an asymmetric pattern. The forms of J and R that produce the A_{1g}-mode of $Cs_2AgFeCl_6$ are

$$J = \begin{pmatrix} 1 & 0 & 0 \\ 0 & e^{0.7i} & 0 \\ 0 & 0 & 1 \end{pmatrix}, R(A_g) = \begin{pmatrix} a & 0 & 0 \\ 0 & a & 0 \\ 0 & 0 & \beta a \end{pmatrix},$$

where β= 0.9 is used to account for the tetragonal distortion. Both J and R percieve tetragonal symmetry. The PO-Raman signal was normalized, and therefore a = 1 was used. We note that

a constant background signal is present in both parallel (||) and perpendicular (⊥) configurations, though this does not alter the physical interpretation of the PO-Raman data.

Figure S2 PO-Raman azimuthal-dependent mapping of the A_g-mode in $Cs_2AgFeCl_6$ at 4K with different *J* and *R* parameters.

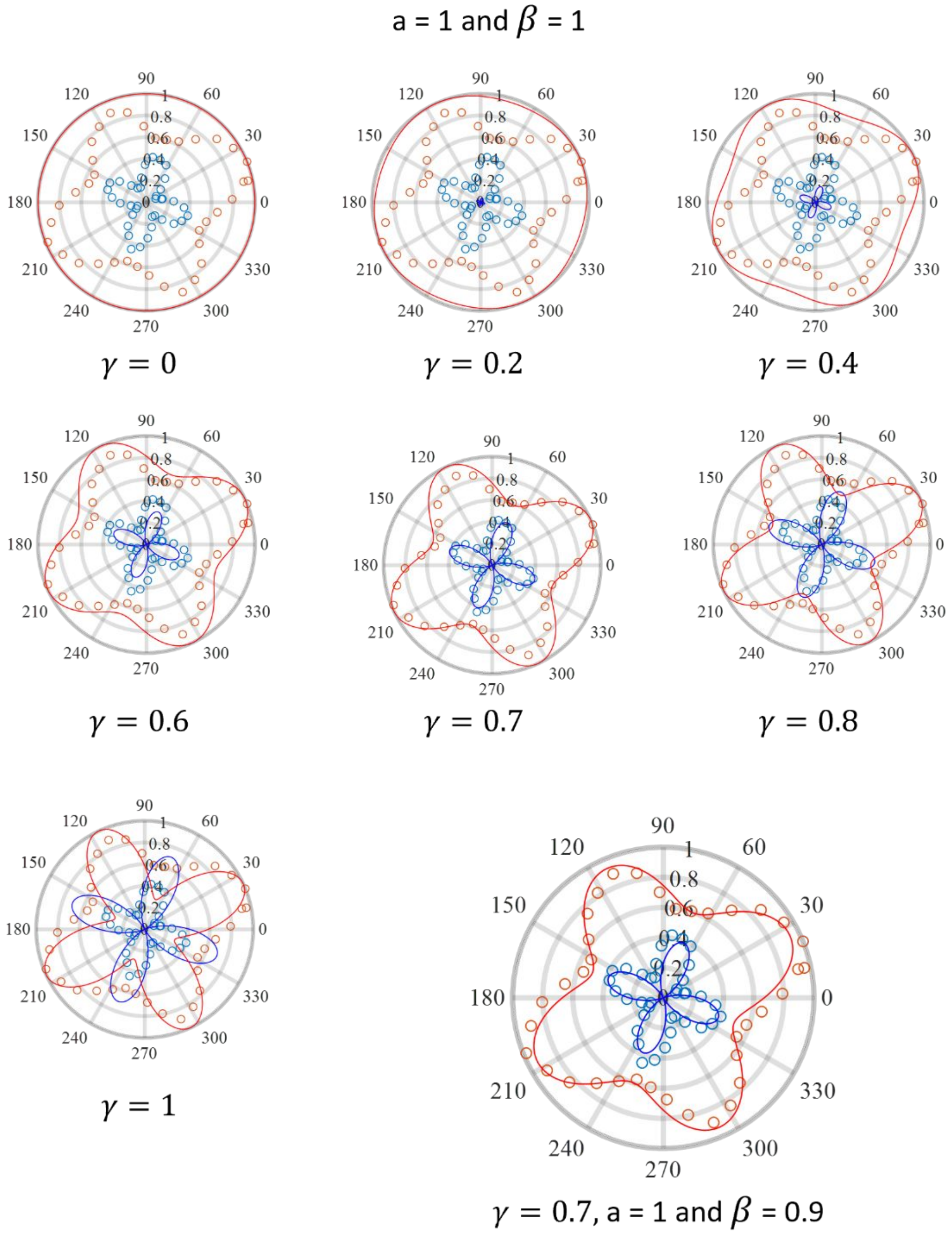

Figure S3A Rietveld plot of the Neutron Powder Diffraction (NPD) data of $Cs_2Ag_{0.6}Na_{0.4}FeCl_6$ (Ag/Na mass concentration ratio) at 1.5K collected on the WISH diffractometer at average 2θ=58.3. **B** The magnetic configuration of $Cs_2Ag_{0.6}Na_{0.4}FeCl_{6,}$ with AFM-I in the $Fm\bar{3}m$ cubic cell. The Ag:Na atomic alloy ratio was confirmed by X-ray diffraction (XRD) from previous work [9].

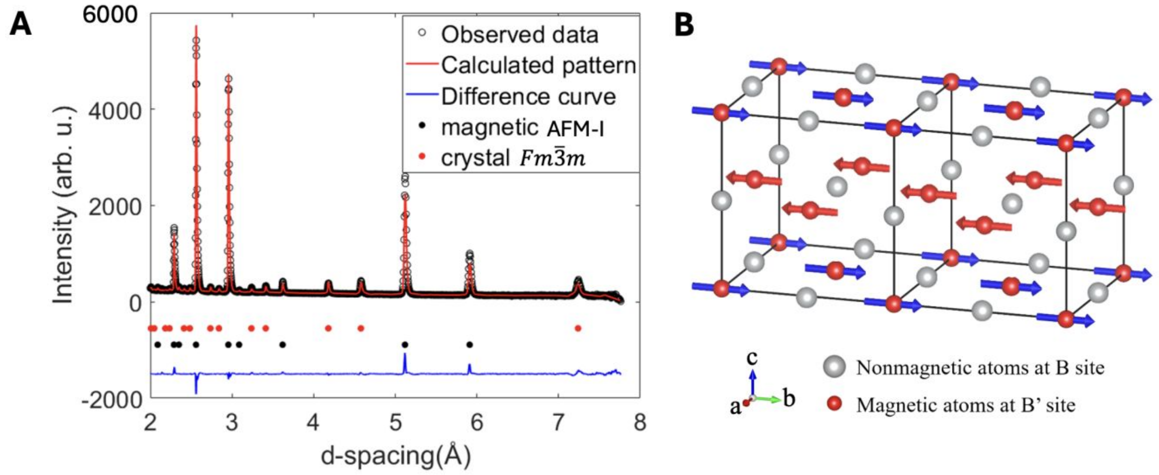

Electronic Structure Calculations

All density functional theory (DFT) calculations were performed using the projector augmented wave (PAW) method as implemented in the Vienna ab initio simulation package (VASP) [1-4]. Geometry optimizations were conducted using the PBEsol functional with Hubbard U correction [5-7]. Structures were relaxed until the residual atomic forces were below10^{-4} eV Å^{-1}. A *k*-point density of 0.15 Å^{-1} and Gaussian smearing width of 0.05 eV were applied. A Hubbard U correction of 3 eV was used throughout.

[1] P. E. Blöchl. Projector augmented-wave method. *Phys. Rev. B* **50**, 17953–17979 (1994).

[2] G. Kresse & J. Furthmüller. Efficiency of ab-initio total energy calculations for metals and semiconductors using a plane-wave basis set. *Comput. Mater. Sci.* **6**, 15–50 (1996).

[3] G. Kresse & J. Furthmüller. Efficient iterative schemes for ab initio total-energy calculations using a plane-wave basis set. *Phys. Rev. B* **54**, 11169–11186 (1996).

[4] G. Kresse & D. Joubert. From ultrasoft pseudopotentials to the projector augmented-wave method. *Phys. Rev. B* **59**, 1758–1775 (1999).

[5] J. P. Perdew. K. Burke. & M. Ernzerhof. Generalized Gradient Approximation Made Simple. *Phys. Rev. Lett.* **77**, 3865–3868 (1996).

[6] A. I. Liechtenstein, V. I. Anisimov, & J. Zaanen. Density-functional theory and strong interactions: Orbital ordering in Mott-Hubbard insulators. *Phys. Rev. B* **52**, R5467–R5470 (1995).

[7] S. L. Dudarev, G. A. Botton, S. Y. Savrasov, C. J. Humphreys & A. P. Sutton. Electron-energy-loss spectra and the structural stability of nickel oxide: An LSDA+U study. *Phys. Rev. B* **57**, 1505–1509 (1998).
